# Magi Is Associated with the Par Complex and Functions Antagonistically with Bazooka to Regulate the Apical Polarity Complex

**DOI:** 10.1371/journal.pone.0153259

**Published:** 2016-04-13

**Authors:** Mojgan Padash Barmchi, Gayathri Samarasekera, Mary Gilbert, Vanessa J. Auld, Bing Zhang

**Affiliations:** 1 Department of Zoology, University of British Columbia, Vancouver, British Columbia, Canada; 2 Department of Biology, University of Oklahoma, Norman, Oklahoma, United States of America; 3 Division of Biological Sciences, University of Missouri, Columbia, Missouri, United States of America; Texas A&M International University, UNITED STATES

## Abstract

The mammalian MAGI proteins play important roles in the maintenance of adherens and tight junctions. The MAGI family of proteins contains modular domains such as WW and PDZ domains necessary for scaffolding of membrane receptors and intracellular signaling components. Loss of MAGI leads to reduced junction stability while overexpression of MAGI can lead to increased adhesion and stabilization of epithelial morphology. However, how Magi regulates junction assembly in epithelia is largely unknown. We investigated the single *Drosophila* homologue of Magi to study the *in vivo* role of Magi in epithelial development. Magi is localized at the adherens junction and forms a complex with the polarity proteins, Par3/Bazooka and aPKC. We generated a *Magi* null mutant and found that *Magi* null mutants were viable with no detectable morphological defects even though the Magi protein is highly conserved with vertebrate Magi homologues. However, overexpression of Magi resulted in the displacement of Baz/Par3 and aPKC and lead to an increase in the level of PIP3. Interestingly, we found that Magi and Baz functioned in an antagonistic manner to regulate the localization of the apical polarity complex. Maintaining the balance between the level of Magi and Baz is an important determinant of the levels and localization of apical polarity complex.

## Introduction

A common component of junctional and polarity complexes is modular scaffolding proteins that are capable of binding to each other as well as recruiting other proteins to the complex. Magi proteins are evolutionarily conserved scaffolding proteins and contain multiple domains including a N-terminal catalytically inactive GUK domain, two WW domains and five to six PDZ (PSD95/Dlg/ZO-1) domains [1]. There are three MAGI proteins in vertebrates (MAGI-1,2,3) all with multiple splice isoforms. MAGI-1 and MAGI-3 are relatively ubiquitously expressed and localize to a range of junctions including epithelial tight junctions. MAGI-2 (also known as AIP1/S-SCAM/ARIP1) is expressed in the nervous system as a synaptic protein and within glomerular podocytes in the kidney [2] and plays important role in scaffolding synaptic proteins such as NMDA receptors and Neuroligin [3], the tip-link protocadherin Cadherin23 [4], the Kir4.1 K(+) channel [5], as well as kidney proteins such as nephrin [6] and JAM4 [7].

Within epithelia and endothelia, MAGI-1 and -3 are localized at tight junctions and form a structural scaffold for the assembly of junctional complexes [8,9]. MAGI-1 also localizes and plays a role in modulating adherens junction adhesion through scaffolding beta-catenin and PTEN [10–12]. MAGI-1 overexpression stabilizes adherens junctions and epithelial cell morphology through increased E-cadherin and β-catenin recruitment [13]. Silencing of MAGI-1 has the opposite effect with decreased adherens junction adhesion and reduced focal adhesion formation leading to anchorage-independent growth and migration *in vitro*. MAGI-1 overexpression suppresses the invasiveness of MDCK cells, as well as suppresses tumor growth and spontaneous lung metastasis through the increased recruitment of PTEN [14] or β-catenin and E-cadherin [13].

Overall, MAGI proteins play important roles in the stabilization of cell—cell interactions and as such Magi is a key target in polarized epithelia during cell death and viral infection. For instance, MAGI-1 is cleaved by activated caspases during apoptosis, a process thought to mediate the disassembly of cell-cell contacts [15]. MAGI proteins are also targeted by a number of oncogenic viruses: it is aberrantly sequestered in the cytoplasm by Adenovirus E4orf1, and is targeted for degradation by the E6 oncoprotein of high-risk human papillomavirus [16]. E6-mediated degradation of MAGI-1 in cultured epithelial cells leads to loss of tight-junction integrity [17–20].

There is a high degree of conservation of protein structure and function in the invertebrate homologues of Magi in particular with regards to epithelial junction formation and maintenance. In *C*. *elegans*, Magi-1 plays a role in the segregation of different cell adhesion complexes into distinct membrane domains along the lateral plasma membrane [21,22]. In *Drosophila*, Magi binds Ras association domain protein 8 (RASSF8) and modulates adherens junctions remodeling in late eye development during interommatidial cell (IOC) rearrangements [23]. In this context Magi function is necessary to recruit the polarity protein Par-3 (*Drosophila* Bazooka, Baz) to the remodeling adherens junction. However, the association of *Drosophila* Magi or any Magi homologue with any components of the Par polarity complex in stable epithelia has not been determined.

The Par complex consisting of Par-3/Par-6/aPKC localizes to tight junctions where MAGI is present in vertebrate epithelial cells and is necessary for assembly of this junctional complex as well as for separation of the apical region of the plasma membrane from the basolateral domain [24–26]. In *Drosophila* epithelial cells, the Par complex localizes to the apicolateral membrane and demarcates the boundary between the apical and basolateral membrane regions. Mutant embryos for any member of this complex show loss of apicobasal polarity and disruption in the integrity of epithelia [27–32]. Although the members of the Par complex are important for the establishment of cell polarity, some of the core components of this complex such as Baz are dispensable for the maintenance of cell polarity during later stages of development. Baz localizes to adherens junction and mutant clones of *baz* in wing imaginal discs are fully viable with no polarity or adherens junction defects. Similarly, Magi function in AJ stability has been determined in many systems, but surprisingly loss of *Drosophila Magi* has no effect on established, stable AJs [23]. Little is known about the convergence of Magi and Par complex function at the adherens junctions and it is possible that Baz and Magi function in established epithelia are redundant. Therefore we investigated the role of Magi in the established and stable epithelia of the *Drosophila* wing imaginal disc to test the potential interactions between Magi and members of the Par complex.

We found that *Drosophila* Magi is associated with the PAR polarity complex and is localized at the adherens junction with Baz, Par-6, and aPKC. Overexpression of Magi resulted in the reduction of apical polarity proteins from the membrane and these interactions required the second half of the Magi protein containing the four PDZ domains. Overexpression of Baz resulted in a reduction of Magi from the membrane but an increase in aPKC and Par-6. While Magi mutants were viable with no polarity defects, we found that Magi levels are antagonistic with Baz and that a balance between the two is necessary to regulate the level and localization of Par complex.

## Material and Methods

### Fly strains and genetics

The following *Drosophila* lines were used: UAS-mCD8::GFP [33], UAS-Magi::Cherry, UAS-aPKC::GFP [34], UAS-Dlg::GFP [35], *aPKC*^*k06403*^FRTG13 [36], *crb*^*11A22*^ FRT82B [37], *sdt*^*EH681*^ FRT19A [38], *baz*^*4*^ FRT9.2 [29], UAS-MagiΔPDZ and UAS-MagiΔW [39], UAS-aPKC-RNAi [40], *Pten*^*dj189*^ FRT13, UAS-Pten a gift from Dr. Andrea Wodarz, Baz::GFP, UAS-Baz::GFP, UAS-P35, apterous-Gal4, FRT9.2Ubi-GFP, FRTG13Ubi-GFP, hsflp FRT19AUbi-mRFP, hs-flipase (on the 2^nd^ chromosome), hs-flipase (on the X chromosome), hs-flipase (on the third chromosome), PH::GFP and Gal80ts were all from the Bloomington *Drosophila* Stock Center.

### Generation of the *Magi*^*bst*^ null mutant

To generate a Crispr/Cas9 mediated deletion of *Magi*, target sequences were selected in the first and last exons of *Magi* gene. The target sequences were 20 nucleotide long followed by a 3 nucleotide PAM, NGG sequence: GCACAGAT|GTGGTTGGTAAC GGG, GGCATCTC|CCAGTCTCCTGA AGG and screened for potential off-target cutting. The targeting ChiRNAs were generated by using the following oligos: 5’CTTCGCACAGATGTGGTTGGTAAC-3’, 5’-AAACGTTACC AACCACATCTGTGC-3’, 5’CTTCGGCATCTCCCAGTCTCCTGA-3’, 5’-AAACTCAGGA GACTGGGAGATGCC-3’, oligos were 5’ phosphorylated using the T4PNK and subsequently annealed by running the following thermocycler program: 95°C for 5 min, then ramp to 25°C at a rate of -5°C/min. The annealed oligos were ligated into the BbsI site in the pU6-BbsI-chiRNA plasmid (a gift from Dr. Kate O'Connor-Giles, University of Wisconsin-Madison) and the resulting purified plasmids sent to Rainbow Transgenic Flies (Camarillo, CA, USA) for injection into the Cas9 expressing embryos. Flies were screened using PCR with primers flanking the expected deletion region and the resulting PCR fragments sequenced to verify the deletion.

### Generation of UAS-Magi::Cherry transgenic line

The full length coding sequence of Magi-RA (LD27118) (DGRC) was amplified by PCR using oligonucleotides HindIIIMagiStart: 5’- AAG CTT ATG CCA ATA ATC ACG GCC AC-3’ and XhoIMagiStop: 5’- CTC GAG TCA TAA TTG TGG CTG CTG ATG-3’ and subcloned into pGEM-T (Promega). The monomeric Cherry coding sequence was obtained (Addgene) and amplified by PCR oligonucleotides EcoRICherryStart: 5’- GAA TTC ATG GTG AGC AAG GGA GAG-3’ and HindIIICherryStop: 5’-AAG CTT CTT GTA CAG CTC GTC CAT G-3’ into pGEM-T. The mCherry fragment was subcloned into pBluescript II (KS+) (Fermentas) using EcoRI and HindIII sites and the Magi-RA into mCherry-Bluescript 3’ to mCherry using HindIII and XhoI sites. The Cherry-Magi 4328 bp fragment was subcloned into pUAST using EcoRI and XhoI. *w*^*1118*^ embryos were transformed by Genetic Services, Inc. (Sudbury, MA, USA) and multiple independent insertions lines created.

### Generation of Magi polyclonal antibody

The Magi polyclonal antibody was generated by immunizing rabbits with a peptide corresponding to the Magi amino acid sequence (ERPSNNISATLALHQQPQL) and subsequent affinity purification both by YenZym Antibodies, LLC.

### Immunohistochemistry

0–20 hrs embryos were collected, dechorionated and fixed by boiling (Cold Spring Harbor Protocols). Wing imaginal discs from third instar larvae were dissected and fixed in 4% formaldehyde. Fixed embryos or wing imaginal discs were washed three times in PBS containing 0.1% Triton-X-100 and blocked with normal goat serum before incubation with primary antibodies. The primary antibodies used were rabbit anti-Magi 1:200 (this study), mouse anti-Crb 1:50 (Cg4, Developmental Studies Hybridoma Bank), rabbit anti-Baz 1:1000 [41], rabbit anti-aPKC zeta C20 1:1000 (Santa Cruz Biotechnology), mouse anti-Dlg 1:50 (Developmental Studies Hybridoma Bank), rabbit anti-Calnexin 1:300 (a gift from I.R. Nabi), rabbit anti-Par6 1:300 [42], mouse anti-GFP 1:500 (Sigma), rabbit anti-cleaved Cas3 1:300 (Cell Signaling), rat anti-DE-cadherin DCAD2 1:50 (Developmental Studies Hybridoma Bank), mouse anti-Flag (M2, Sigma), mouse anti-GFP (Sigma), rat anti-Magi [43], mouse anti-Arm (Developmental Studies Hybridoma Bank). For visualizing lipid rafts biotin conjugated Cholera toxin B (CTB) (Life Technologies) 1:300 was used and subsequently detected with StreptavidinA647 at 1:100. The appropriate secondary antibodies were conjugated Alexa488, Alexa594, and Alexa 647 (Invitrogen).

Proximity Ligation Assays were carried according to the manufacturer’s specifications (Duolink, Sigma). Prior to PLA, wing imaginal discs were isolated and fixed as outlined above then incubated with primary antibodies at 4°C overnight. After washes in PBS+0.1% Triton, 3 times for 15 minutes, discs were incubated in PLA probe solution at 37°C for 1.5 hours. Two washes for 5 minutes in Wash A solution were followed by the Ligation reaction at 37°C for 1 hour, two washes for 2 minutes and then the Amplification reaction at 37°C for two hours. Discs were washed in Wash B two times for 10 minutes and mounted in Vectashield.

Images were collected using an Olympus FV1000 confocal microscope and a Plan-Apochromat 60x oil (1.42 NA) objective or DeltaVision Spectris microscope (Applied Precision, Issaquah, WA) with a 60X (1.42 NA) oil immersion lens using a CoolSnap HQ digital camera. Low magnification images were collected on a Zeiss Axioskop with a 20x NA 0.50 air lens using Northern Eclipse Software 8.0. For Deltavision images, SoftWorx (Applied Precision) software was used for deconvolution of 10–15 iterations using a point-spread function calculated with 0.2 micron beads conjugated with Alexa568 (Molecular Probes) mounted in Vectashield. For Deltavision data analysis, images were processed using SoftWoRx software and all other data were analyzed using ImageJ (NIH). Figures were made using Adobe Photoshop software. For quantification of membrane levels of proteins, the florescent intensity at the membrane was measured for a constant ROI in overexpression versus non-overexpression sides using the “find edges” function of ImageJ followed by threshold adjustments and measurement of intensity. The data were transferred to Excel for statistical analysis using an unpaired t-test and graph creation.

### Immunoprecipitation and Western blotting

For immunoprecipitations, overnight collection of Baz::GFP embryos were dechorionated and lysed in lysis buffer (10mM Hepes, 0.1mM MgCl2, 150mM NaCl, 5mM NEM, 2mM PMSF, 1% Triton X-100 and protease inhibitor cocktail (Roche)). After centrifugation 10μl of purified Magi antibody (0.36mg/ml) was added to cell lysate containing 1.5 mg of total protein. Immunocomplexes were harvested using 100 μl of protein-A microbeads (Miltenyi Biotech) and washed four times in low salt buffer (150mM NaCl, 50mM Tris-HCl pH 8.0, 1%NP-40, protease inhibitor cocktail EDTA free (Thermo Scientific)). Bound proteins were eluted in pre-heated SDS buffer and were subjected to electrophoresis and western blotting. Western blots were probed with rabbit anti-Magi 1:1000, rabbit anti-Baz 1:2000, rabbit anti-aPKC zeta C20 1:2000 and mouse anti-Dlg 4F3 1:500. To detect the primary antibodies HRP-conjugated goat anti-mouse (1:4000) and HRP-conjugated goat anti-rabbit (1:4000) secondary antibodies were used (JacksonImmunolabs). The HRP signal was detected using ECL Plus (GE Healthcare) and imaged on a ChemiDoc XRS+ imaging system (BioRad). For analysis of *Magi* mutants, proteins were isolated from overnight collections of wildtype embryos, homozygous *Magi* mutant embryos or approximately 40 third instar wing imaginal discs from wildtype and *Magi* mutants. Samples were extracted in loading buffer, and subjected to gel electrophoresis and western blotting. Western blots were probed with rabbit anti-Magi (this study) 1:1000 and anti -Tubulin (1:2000) as an internal loading control.

## Results

### Magi colocalizes with the apical polarity complex and the adherens junction

**Fig 1:**
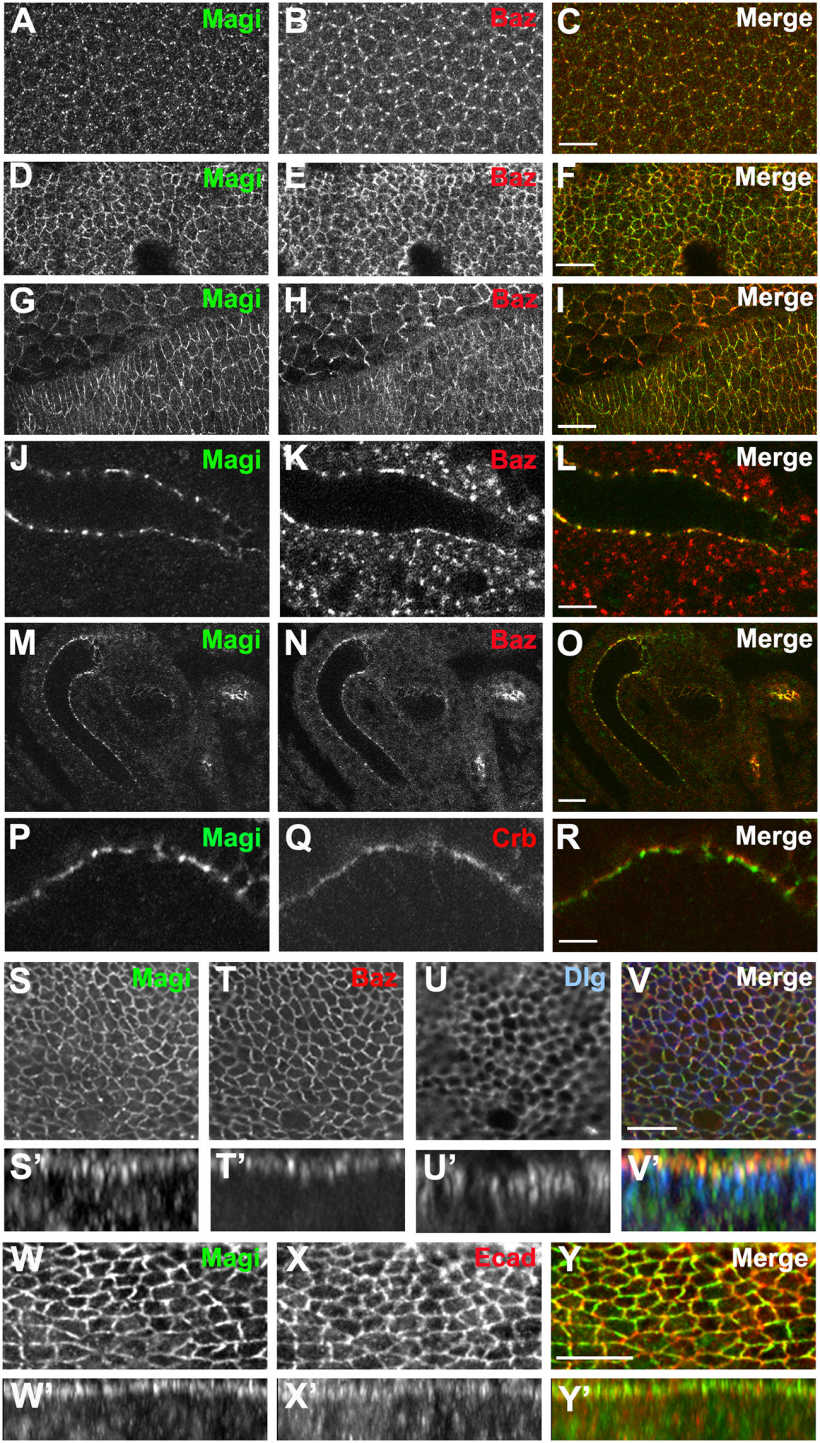
Magi localizes to the apicolateral region and adherens junctions in epithelia. (A-C). Enface view of the cellularizing embryo showing Magi (green) localization to the furrow canal where Baz (red) is present. (D-F). Enface view of stage 11 embryo showing Magi (green) present in the same membrane domain as Baz (red). (G-I). Enface view of stage 13 embryo showing Magi (green) is present in the same membrane domain as Baz (red) in both epithelial and amnioserosa cells. (J-L). Side view of the salivary gland showing Magi (green) and Baz (red) colocalizing in the same membrane region at the apicolateral domain. (M-O). Side view of the hindgut showing Magi (green) and Baz (red) colocalization at the apicolateral membrane. (P-R). Side view of the salivary gland showing Magi (green) is below the apical membrane domain where Crumbs (Crb, red) is present. (S-V’). Enface and side views of wing imaginal disc epithelia showing that Magi (green) predominantly colocalizes with Baz (Baz::GFP, red) but not with Dlg (blue). (W-Y’). Enface and side view of the wing imaginal disc epithelia showing that Magi (green) is present at the adherens junction, marked with Ecad (red) immunolabeling. Scale bars indicate 10μm. Each enface view represents a single Z slice.

*Drosophila* has a single Magi homologue that contains four PDZ domains, two WW domains but lacks the GUK domain (S1 Fig). As a first step to understanding the role of Magi, we investigated the distribution and role of Magi in polarized epithelia. We raised an antibody against Magi (S1 Fig) and found that Magi was localized to the apico-lateral membrane in embryonic and wing imaginal disc epithelia (Fig 1). During embryonic development Magi is present very early during cellularization and colocalizes with Bazooka (Baz, *Drosophila* Par-3) at the furrow of the forming polarized cells (Fig 1A–1C). Magi remains colocalized with Baz at the apicolateral membrane after cellularization and throughout embryonic development in all stages (Fig 1D–1O). Immunolabeling analysis of Magi and the apical protein Crumbs (Crb) revealed that Magi was at the apicolateral membrane domain below the marginal zone where Crb is present (Fig 1P–1R). In the columnar epithelia of the wing imaginal disc, Magi also colocalizes with Baz but it is excluded from the basolateral domain and does not overlap with Discs-large (Dlg) (Fig 1S–1V). We examined the localization of Magi with respect to the adherens junction protein E-cadherin (Ecad) and found that Magi colocalizes with Ecad (Fig 1W–1Y).

**Fig 2:**
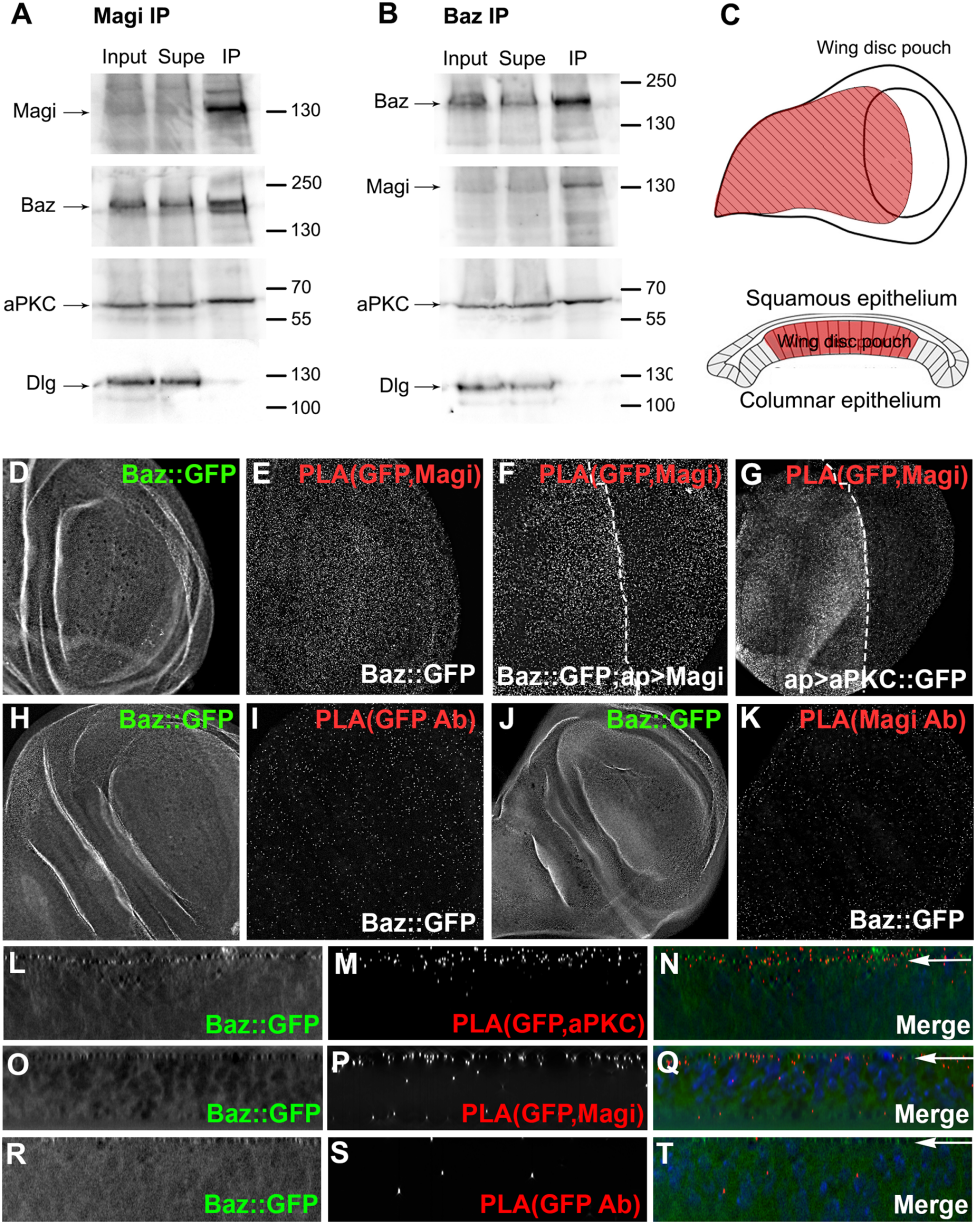
Magi associated with Baz and aPKC at the adherens junctions. (A, B) Immunoprecipitation of embryonic extracts with endogenous Baz tagged with GFP. Proteins from the embryonic extracts (Input), supernatant (Supe) and immunoprecipitation (IP) were loaded for Western analysis. The Magi antibody (A) and the Baz antibody (B) were used for immunoprecipitation and in both experiments Magi is pulled down with Baz and aPKC but not with the basolateral protein Dlg. (C) Diagram of the wing imaginal disc from a third instar larvae with the expression pattern of apterous-GAL4 shown in red and bisecting the wing pouch. A side view shows the wing disc within the columnar epithelia of the wing pouch labeled in red. (D-T) Proximity Ligation Assays were performed on wing imaginal discs with Bazooka endogenously tagged with GFP (Baz::GFP). (C-E) Baz::GFP (green) with PLA (red) using anti-GFP and anti-Magi antibodies. (F) Magi overexpressed with apterous-GAL4 with Baz::GFP with PLA using anti-GFP and anti-Magi antibodies shows greater levels of PLA on the apterous (left) side of the wing disc. (G) aPKC::GFP overexpressed with apterous-GAL4 with anti-GFP and anti-Magi antibodies shows greater levels of PLA on the apterous side of the wing disc. (H-K) Controls with Baz::GFP and PLA (red) with only anti-GFP (G-H) and Baz::GFP and PLA (red) with anti-Magi alone (I-J). PLA levels are reduced compared to panel D. (L-N) Side projection of Baz::GFP (green) with PLA (red) with anti-GFP and anti-aPKC antibodies with the PLA signal concentrated at the apical domain of the columnar epithelium (arrow). Nuclei were labeled with DAPI (blue). (O-Q) Side projection of Baz::GFP (green) with PLA (red) with anti-GFP and anti-Magi antibodies with the PLA signal concentrated at the apical domain of the columnar epithelium (arrow). Nuclei were labeled with DAPI (blue). (R-T) Controls showing a side projection of Baz::GFP discs with PLA using only anti-GFP (R), only anti-Magi (S) or only anti-aPKC (T).

To determine if Magi was associated with the Par-3 complex, we performed immunoprecipitation analysis using protein extracts of embryos in which endogenous Baz was tagged with GFP (Baz::GFP). We used a Magi polyclonal antibody to pull down Magi and any bound proteins and found that Magi associated strongly with Baz and aPKC (Fig 2A), weakly with Par-6 and Crb (data not shown), and not at all with the basolateral protein Dlg (Fig 2A). In order to ensure the specificity of this interaction we carried out similar immunoprecipitation analysis by using a Baz antibody and found that Baz was present in the same complex as Magi and aPKC but not Dlg (Fig 2B). We expanded our analysis to the epithelia of wing imaginal discs and analyzed the association of Magi with the polarity complex and adherens junctions using a proximity ligation assay, which detects protein interactions occurring within 40 nm [44]. A strong PLA signal was observed between Magi and Baz endogenously tagged with GFP (Baz::GFP) (Fig 2D and 2E) compared with control PLA reactions in which only one antibody was used (Fig 2H, 2I, 2J, 2K and 2R–2T). The PLA signal between Magi and Baz::GFP was increased when Magi was overexpressed using apterous-Gal4 (Fig 2F), which is expressed in the dorsal half of the wing imaginal disc (Fig 2C). We observed an increase in the positive PLA signal between Magi and aPKC when aPKC::GFP was driven by apterous-GAL4 (Fig 2G). The PLA signal was concentrated within the apical domain of the columnar epithelia for Magi and Baz::GFP (Fig 2O–2Q), similar to the control PLA between Baz::GFP and aPKC (Fig 2L–2N), while the background PLA was randomly dispersed in controls (Fig 2R–2T). These results suggest that Magi is in close proximity (within 40 nm) with the members of the polarity complex at the adherens junction domain in epithelia of embryos and third instar imaginal discs.

### Magi protein localization depends on aPKC, but not Crb or Baz

**Fig 3:**
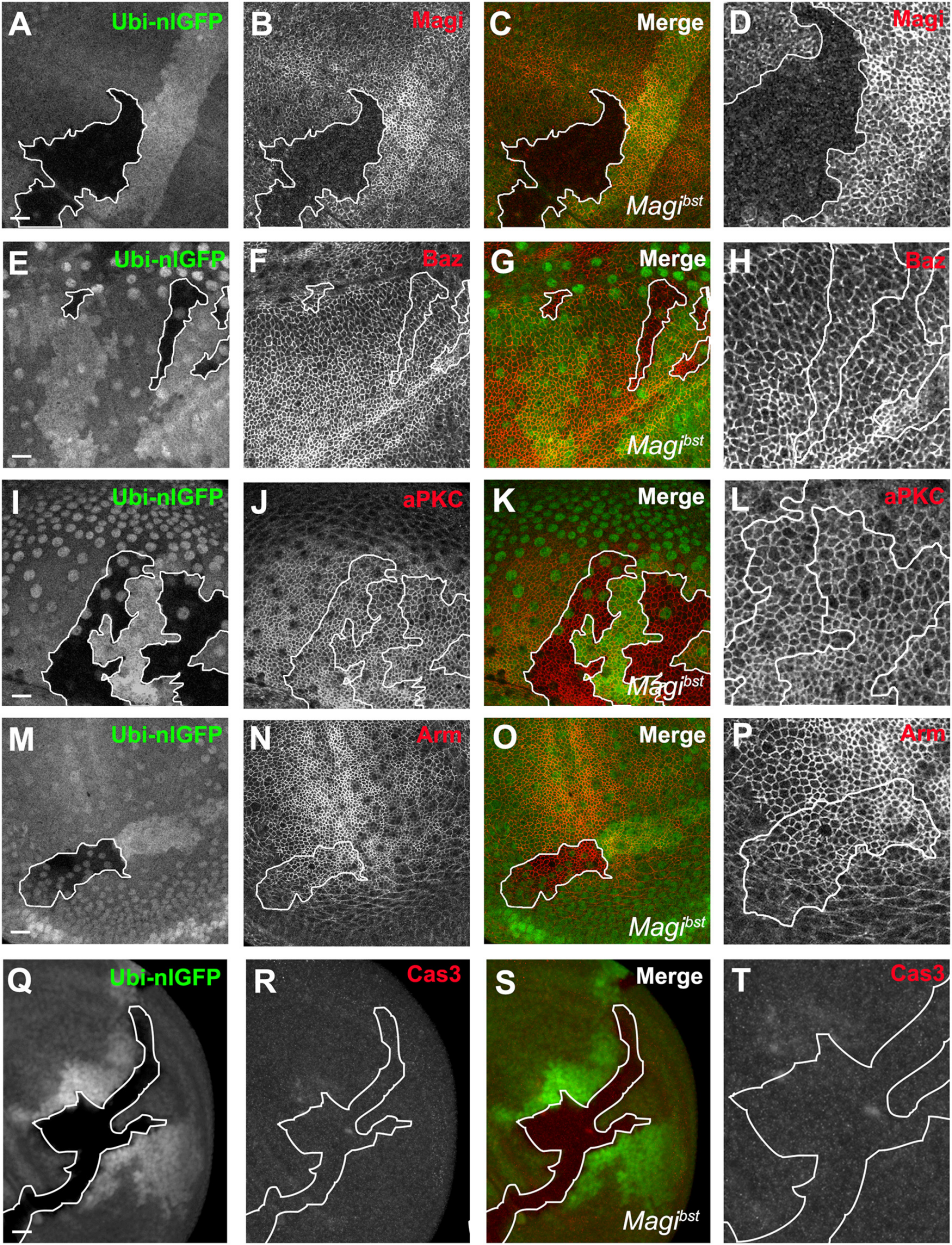
Somatic null clones of *Magi* are viable with no loss of polarity or AJ. Mutant clones of *Magi*^*bst*^ in the wing imaginal disc were generated by FRT mediated recombination. Nuclear-localized GFP driven by the ubiquitin promoter (Ubi-nlGFP, green) labeled wildtype and heterozygous cells, and *Magi*^*bst*^ clones are indicated by the lack of GFP. The large GFP positive nuclei are from the overlying peripodial cells. *Magi*^*bst*^ clones lack Magi immunolabeling (A-D) but had no effect on the levels and localization of Baz (E-H, red), aPKC (I-L, red) or the adherens junction protein Armadillo (Arm, red) (M-P). There was no effect on cell survival, which is shown by lack of apoptotic marker cleaved Caspase-3 (Cas3, red) (Q-S). Panels D,H,L,P,T were digitally magnified 200% and the clonal boundaries are marked with white lines. Scale bars indicate 10μm.

The colocalization and co-immunoprecipitation of Magi with the apical polarity complex prompted us to investigate whether Magi plays a role in epithelial polarity. Using CRISPR/Cas9 [45–48] we created a deletion allele of Magi, named *Magi*^*bst*^, that removed the majority of the *Magi* coding sequence (confirmed by sequencing) and resulted in a loss of Magi immuno-reactivity in both Western blots and in the wing imaginal disc (Fig 3, S1 Fig). The Magi anti-sera also cross-reacted with a non-specific nuclear epitope that was still present in our *Magi* mutants. While loss of Magi was complete, the deletion allele was homozygous viable with no obvious embryonic, larval or adult phenotypes and somatic clones were viable (Fig 3). To explore the functional interaction between Magi and the apical polarity proteins, we examined the localization and levels of components of the apical polarity complex in somatic clones of the *Magi* mutant. Loss of Magi had no effect on the localization and levels of expression of proteins comprising the apical polarity complex including Baz (Fig 3E–3H), aPKC (Fig 3I–3L) and did not affect the adherens junction (Fig 3M–3P) or trigger apoptosis as measured by immunolabeling for activated Caspase 3 (Fig 3Q–3S). These results were surprising given the strong amino acid conservation of the sole Magi in insects with vertebrate Magi. However, other loss of function *Magi* mutants in flies were also found to be viable [23], suggesting that Magi function might be in part redundant with other components of the polarity complex.

**Fig 4:**
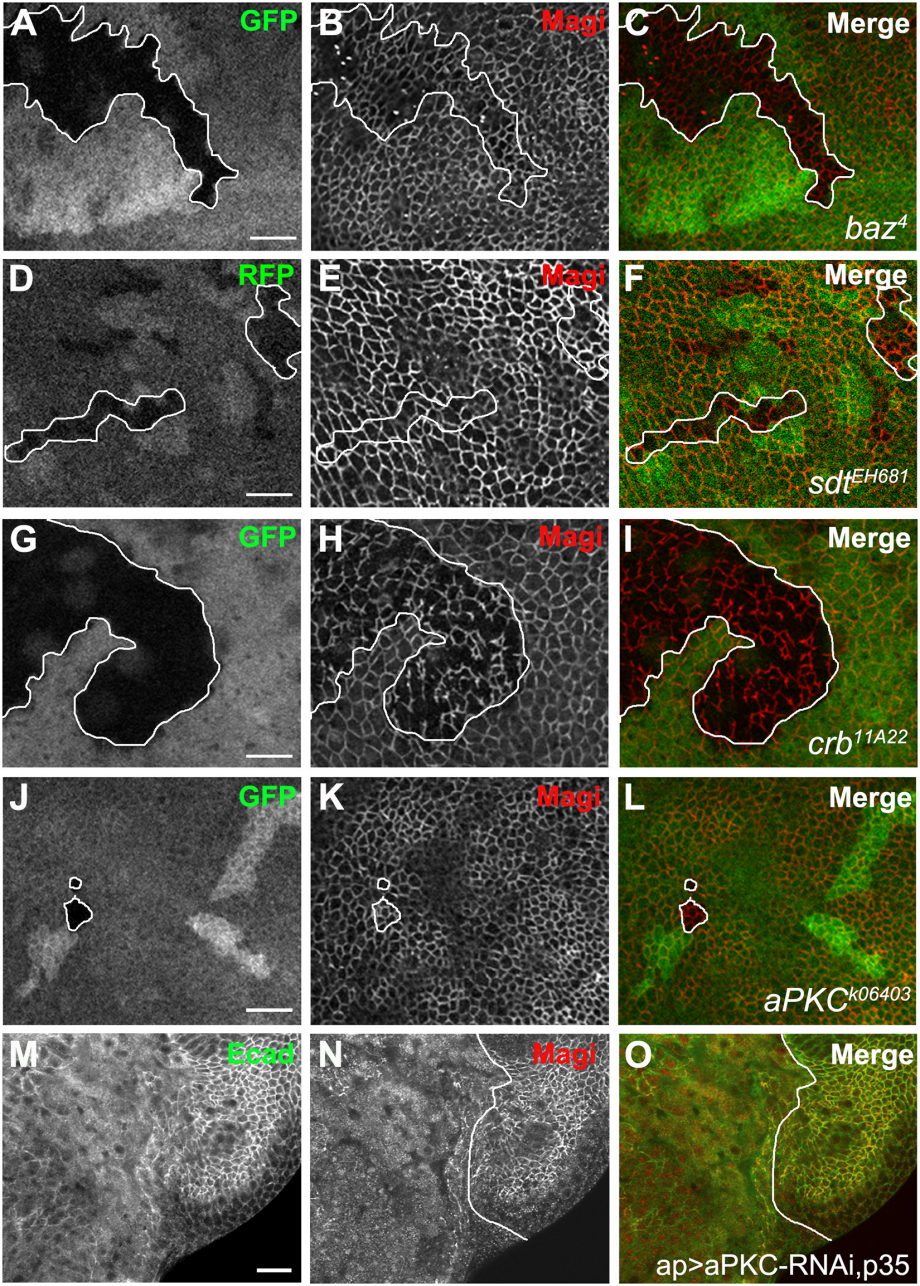
Magi localization requires aPKC but not the other polarity proteins, Baz, Sdt or Crb. (A-I) Wing imaginal discs showing mitotic clones mutant for apical polarity protein genes, *baz*, *sdt* and *crb* (clone boundaries are indicated by white lines). Mutant clones for *baz*, *sdt* and *crb* survive and no polarity defects are seen. Somatic clones are marked by the loss of a cellular marker (GFP or RFP). Loss of these polarity proteins had no effect on membrane recruitment of Magi (red). In *crb* mutant clones, the continuous localization of Magi on the plasma membrane was altered. (J-L) Wing imaginal disc showing mitotic clones mutant for *aPKC*. Only small *aPKC* mutant clones were recovered and frequently twin spots were not associated with mutant clones. Magi (red) did not appear to be affected in the small *aPKC* mutant clones. (M-O) Wing imaginal discs co-expressing aPKC-RNAi and p35 to block cell death (*ap>aPKC-RNAi*, *p35*). Temporal control of expression was controlled using Gal80ts. Blocking cell death resulted in tissue overgrowth and loss of apicobasal polarity as Ecad (green) localization was impaired. Magi (red) localization to the plasma membrane was also disrupted. Scale bars indicate 5μm in A-L and 10μm in M-O.

In order to determine whether the recruitment of Magi to the membrane and polarity complex requires the function of the apical polarity protein complex, we examined the localization and level of Magi in somatic mutant clones of the core components of the apical polarity complexes *baz*, *crb*, *sdt*, and *aPKC*. Our results show that loss of *baz* and *sdt* had no effect on Magi levels or its localization at the plasma membrane (Fig 4A–4F). Loss of *crb* altered the continuous localization of Magi at the membrane but it did not affect the actual recruitment of Magi to the plasma membrane (Fig 4G–4I). These results suggest that Magi recruitment to the polarity complex is not dependent on the functions of the core components of the apical polarity complexes. Somatic clones of *baz* do not disrupt the polarity of wing disc epithelia suggesting that Baz and Magi might be redundant. However, we found that somatic clones of a *baz* null mutant in a *Magi* mutant background did not lead to a loss of cell polarity or apoptosis (S2 Fig). We were unable to clearly determine the localization of Magi in *aPKC* clones (Fig 4J–4L), which are small due to cell lethality [31,36,49]. In order to circumvent this problem, we expressed aPKC-RNAi in the wing imaginal disc and simultaneously blocked cell death by expressing the baculoviral protein, p35 [50]. Blocking apoptosis resulted in tissue overgrowth as well as the loss of cell polarity indicated by the loss of Ecad at the adherens junction (Fig 4M). Under these conditions Magi levels were reduced in the plasma membrane (Fig 4N), suggesting either that aPKC is necessary for recruitment of Magi to the membrane or that loss of Magi is a secondary effect to the loss of apical basal polarity.

### Magi overexpression reduces membrane localization of apical polarity proteins

**Fig 5:**
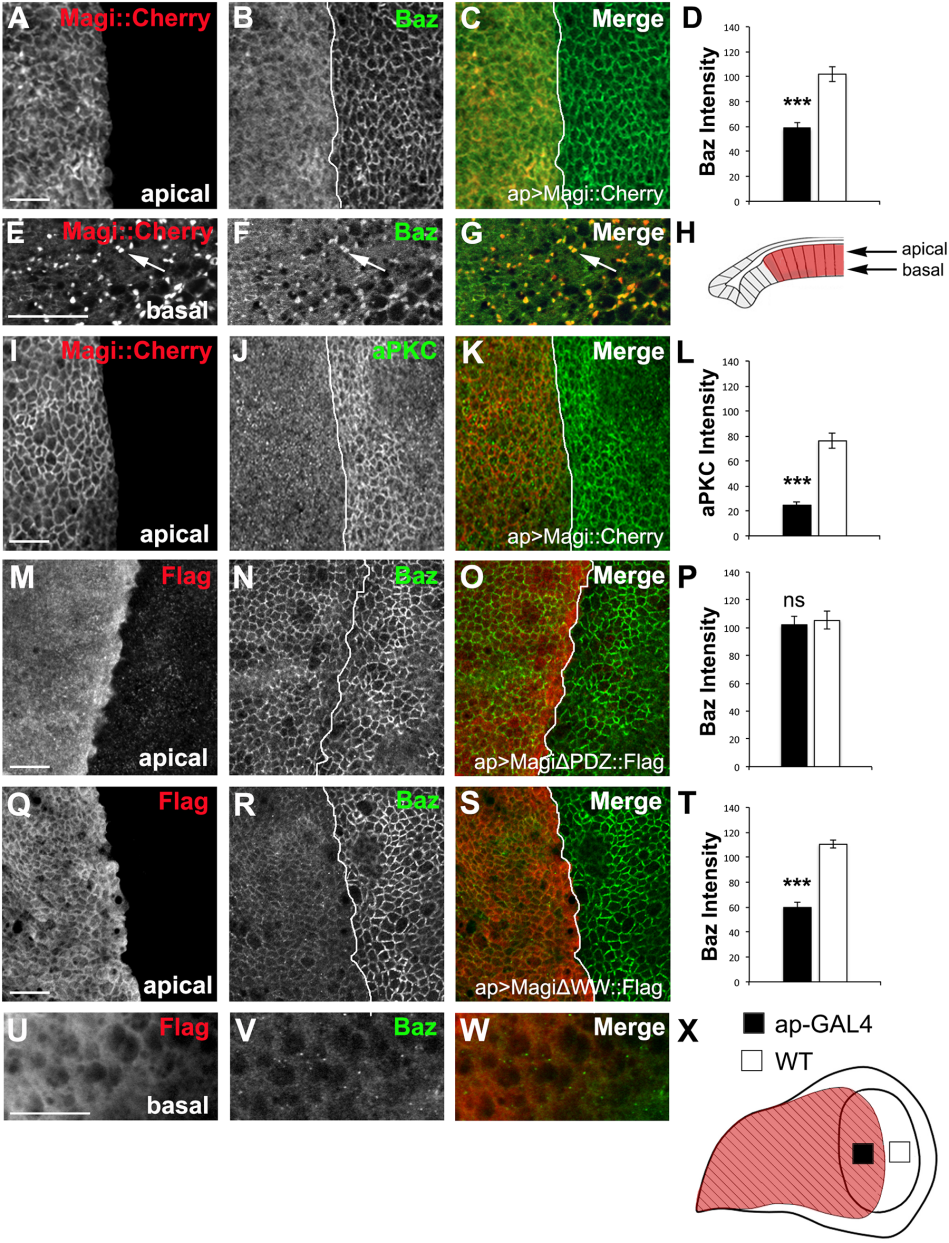
Overexpression of Magi reduces Baz at the plasma membrane. Cherry-tagged Magi was overexpressed in the wing imaginal disc using apterous-GAL4 (ap>Magi::Cherry). The average membrane intensity of Baz, and aPKC was measured and compared between the apterous (ap-GAL4, black bars) and non-apterous side (WT, white bars) of the wing imaginal disc (X). The apterous boundary is indicated with a white line and in all panels the apterous side is to the left. All panels represent a single Z slice within the apical or basal domain of the columnar epithelia (H). (A-D) Wing imaginal disc overexpressing Magi::Cherry (red) immunolabeled for Baz (green). High levels of Magi result in mislocalization of Baz to the cytoplasm and a significant reduction on the plasma membrane (D). (E-G) At the basolateral region of the epithelia (H), Baz (green) was detected in large puncta (arrows) with Magi::Cherry (red). (I-L) Wing imaginal discs overexpressing Magi::Cherry (red) immunolabeled for aPKC (green). aPKC was significantly reduced at the plasma membrane (I) and present in small cytosolic puncta. (M-P) Wing imaginal disc expressing a Magi transgene lacking the PDZ domains (MagiΔPDZ) tagged with the FLAG epitope (red). Expression of this transgene had no effect on the membrane localization of Baz (green)(P) and the Magi protein appeared to be cytosolic. (Q-T) Wing imaginal disc expressing a Magi transgene lacking the two WW domains (MagiΔWW) tagged with the FLAG epitope (red). Expression of this transgene resulted in a significant reduction in Baz (green)(T). (U-W) MagiΔWW was found at the membrane but did not form large intracellular accumulations in basal regions. *** p<0.001; ns p>0.05. n = 5 discs for each experiment and error bars indicate SEM. Panels (E-G, U-W) were digitally magnified 200%. Scale bars indicate 5μm.

To test the effects that overexpression of Magi would have on the apical polarity complex, we constructed a transgene with Magi tagged at the C-terminal domain with Cherry RFP (Magi::Cherry). Overexpression of Magi in the wing imaginal disc epithelia using *apertous*-Gal4 (*ap*) resulted in a reduction of the apical polarity complex at the plasma membrane (Fig 5). Baz was reduced at the apical plasma membrane and found in a more diffuse pattern within the cytoplasm (Fig 5A–5D). In basal regions within the cells Baz also appeared concentrated in large intracellular accumulations together with Magi (Fig 5E–5G). aPKC was dramatically reduced at the plasma membrane and was found in small cytoplasmic puncta (Fig 5I–5L). To a lesser extent Par-6 and Crb were reduced at the membrane and were redistributed into the cytoplasm (S3 Fig). In contrast, overexpression of Magi had no effect on the localization or the levels of the adherens junction protein E-Cad or the basolateral polarity protein Dlg (S3 Fig). Baz was the only polarity protein found colocalized to the large internal Magi accumulations; we were unable to detect Crb, Par-6, aPKC, Dlg, or Ecad in these structures (S3 Fig).

The WW and PDZ domains of Magi have been implicated in membrane and protein interactions [1,7,14,51]. Hence, we asked whether these domains of Magi are necessary for the alteration in the level of Baz at the plasma membrane. Expression of a Magi transgene lacking all four PDZ domains (MagiΔPDZ) [39] was poorly recruited to the plasma membrane and did not alter Baz levels (Fig 5M–5P). In contrast, a Magi transgene containing the PDZ domains but lacking the two WW domains (MagiΔWW) [39] was still recruited to the membrane and reduced Baz levels (Fig 5Q–5T). These results suggest that the PDZ domains of Magi or a region in the second half of the protein are essential for Magi recruitment into the plasma membrane and for displacement of Baz from the membrane when Magi is overexpressed. However the MagiΔWW protein did not lead to the accumulation of Magi or Baz within large vesicles (Fig 5U–5W) suggesting that the WW domain or N-terminal half may mediate the accumulation of Magi. Overall these results indicate that overexpression of Magi affects a subset of apical polarity proteins and that this interaction occurs through the PDZ domains of Magi.

### Magi colocalizes with ER and lipid markers

**Fig 6:**
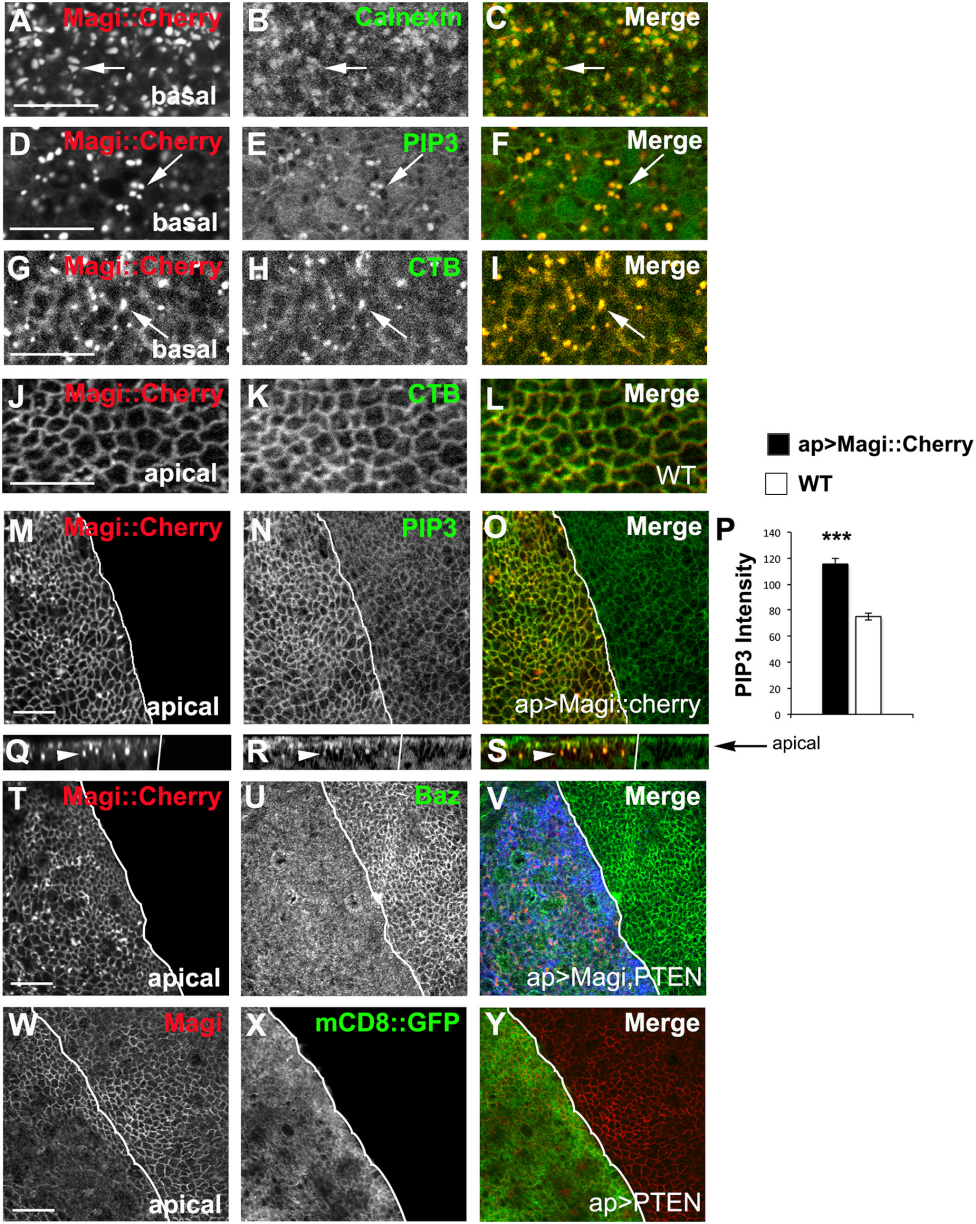
Magi is associated with lipid subdomains and alters PIP3 levels. (A-I) Cherry-tagged Magi (red) was expressed using apterous-GAL4 and accumulations within the basolateral region. (A-C) Magi::Cherry accumulations were positive for the ER marker Calnexin (green), arrows. (D-F) PIP3 (PH::GFP; green) was co-localized in the large accumulations (arrows) with Magi::Cherry. (G-I) Magi::Cherry accumulations were positive for Cholera toxin B (CTB, green)(arrows). (J-L) Endogenous Magi (red) colocalized with cholera toxin B (CTB, green) in wildtype discs at the plasma membrane. (M-S) Cherry-tagged Magi was overexpressed using apterous-GAL4 (ap>Magi::Cherry). The apterous boundary is indicated with a white line. (M-P) Wing imaginal discs overexpressing Magi::Cherry (red) with a PIP3 indicator (PH::GFP, green) under the control of the tubulin promoter. High levels of Magi resulted in an increase in of PIP3 at the plasma membrane. *** p<0.001; n = 5 discs. Error bars indicate SEM. (Q-S) Side projections showing the increase in PIP3 is within the apical domain (arrow) and within the large Magi accumulations (arrowhead). (T-Y) PTEN was expressed using apterous-GAL4 with Magi::Cherry (T-V) or without (W-Y). (T-V) Baz (green) was still displaced from the membrane when PTEN (blue) and Magi::Cherry (red) were coexpressed. (W-Y) Expression of PTEN alone had no effect on Magi recruitment to the membrane (arrowhead). Panels (A-L) were digitally magnified 200%. Scale bars indicate 5μm

The presence of Magi in large accumulations was of interest and we next investigated if these corresponded to known intracellular organelles. The Magi accumulations were not positive for LavaLamp (Golgi), Mito::GFP (mitochondria), Hrs (endosomes), Rab11 (recycling endosomes), LAMP (lysosomes), and Sec8 (ESCRT complex) (data not shown). However, these accumulations were positive for Calnexin (Fig 6A–6C), which suggests retention of Magi at the ER. The accumulations were also positive for a PIP3 marker (Fig 6D–6F), measured using a fusion of GFP with the PH domain of Grp1 that binds specifically to PtdIns(3,4,5)P3 (PIP3) [52]. These results suggest that Magi accumulates in a lipid subdomain or altered the lipid domain. To further test for localization to lipid domains, we used cholera toxin B (CTB) conjugated to a fluorophore, as CTB effectively binds GM1 and other gangliosides/sphingolipids that are enriched in lipid rafts [53–55]. While insects lack GM1 [56], *Drosophila* cells mediate uptake of CTB [57] and *Vibrio cholera* infection [58] likely due to the binding of CTB to other sphingolipids. The Magi accumulations were also strongly positive for CTB binding (Fig 6G–6I). We also observed that in wildtype discs, Magi and CTB were colocalized at the level of the adherens junction (Fig 6J–6L). Our results suggest that the accumulation of Magi is associated with specific subdomains of the lipid environment.

### Magi expression alters PIP3 levels

The association of Magi and lipid markers was of interest given the effect of overexpressed Magi on Baz membrane levels as the recruitment and presence of Baz at the plasma membrane is intimately tied to the levels of PtdIns(4,5)P2 (PIP2). Baz directly binds phosphoinositides and loss of phosphoinositides from the cell membrane alters Baz localization [59–61]. We next investigated whether overexpression of Magi altered the levels of PIP2/3. Overexpression of Magi increased the level of PIP3 at the plasma membrane on the apterous side (Fig 6M–6P) and these increases were within the apical domain (Fig 6Q–6S). These results suggest that changes to Magi protein levels may affect the fine-tuning of PIP2/3 at the membrane domain and thus lead to changes in the levels of Baz. To test if the increase in PIP3 levels was the cause of the Baz displacement, we coexpressed the PIP3 phosphatase PTEN along with Magi::Cherry. The presence of PTEN along with Magi did not block the downregulation of Baz from the membrane (Fig 6T–6V). PTEN expression alone had no effect on Magi localization (Fig 6W–6Y). Similarly somatic clones of *Pten* loss of function mutant had no effect on the recruitment of Baz or Magi to the membrane (S4 Fig). Therefore the loss of Baz from the membrane is not as a result of Magi expression changing the PIP2/3 lipid environment within the apical domain. However it is possible that the loss of Baz from the membrane could lead to increases in PIP3 that we observed given the ability of Baz to recruit PTEN [62].

### Competition between Magi and Baz and their opposite effects on aPKC levels

**Fig 7:**
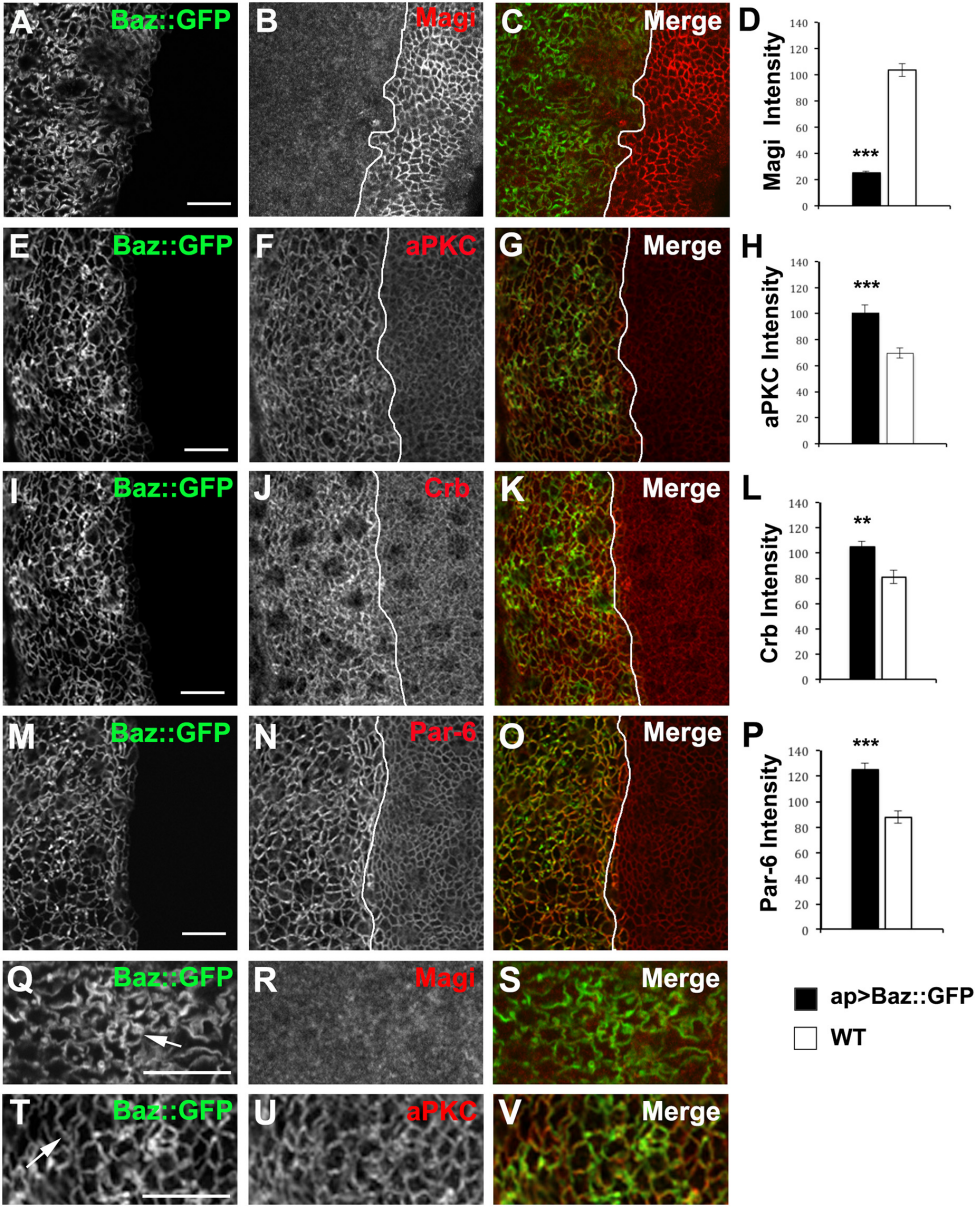
Overexpression of Baz results in loss of Magi from the plasma membrane. GFP-tagged Baz was overexpressed in the wing imaginal disc using apterous-GAL4 (ap>Baz::GFP). The average membrane intensity of each protein was measured and compared between the apterous (black bars) and non-apterous side (white bars) of the wing imaginal disc. The white lines mark the apterous dorsal/ventral boundary. All images showing the apical region of the epithelia. (A-D) Overexpression of Baz::GFP (green) immunolabeled for Magi (red). Cells containing high levels of Baz show a significant reduction in the membrane levels of Magi. (E-P) Overexpression of Baz::GFP (green) resulted in a significant increase in the membrane levels of aPKC (red) (E-H), Crb (red) (I-L) and Par-6 (red) (M-P). (Q-V) Overexpression of Baz::GFP (green) lead to membrane ruffling (arrows) but not the accumulation of Magi (red) or aPKC (red) in intracellular puncta. *** p<0.001; ** p<0.01. n = 5 discs for each experiment and error bars indicate SEM. Panels (Q-V) were digitally magnified 200%. Scale bars indicate 5μm

As overexpression of Magi reduced the plasma membrane association of Baz and aPKC, we tested if overexpression of Baz or aPKC affected Magi. We observed that Baz overexpression resulted in a loss of Magi from the cell membrane (Fig 7A–7D and 7Q–7S). To determine if the effect of Baz overexpression is specific to Magi, we examined the localization and levels of the other components of the apical polarity complex. Interestingly, the levels of aPKC (Fig 7E–7H), Crb (Fig 7I–7L) and Par-6 (Fig 7M–7P) were increased at the plasma membrane in the presence of increased Baz. In contrast, high levels of Baz had no effect on the basolateral polarity protein Dlg (S4 Fig), suggesting that the effect of high levels of Baz at the membrane is specific to the apical polarity complex. Overexpression of Dlg had no effect on Magi localization or levels at the membrane (S4 Fig). We also noted that overexpression of Baz lead to “ruffles” or “contortions” in the plasma membrane (Fig 7Q–7V, arrows) with the Baz expressing cells appearing consistently larger on the apterous side.

**Fig 8:**
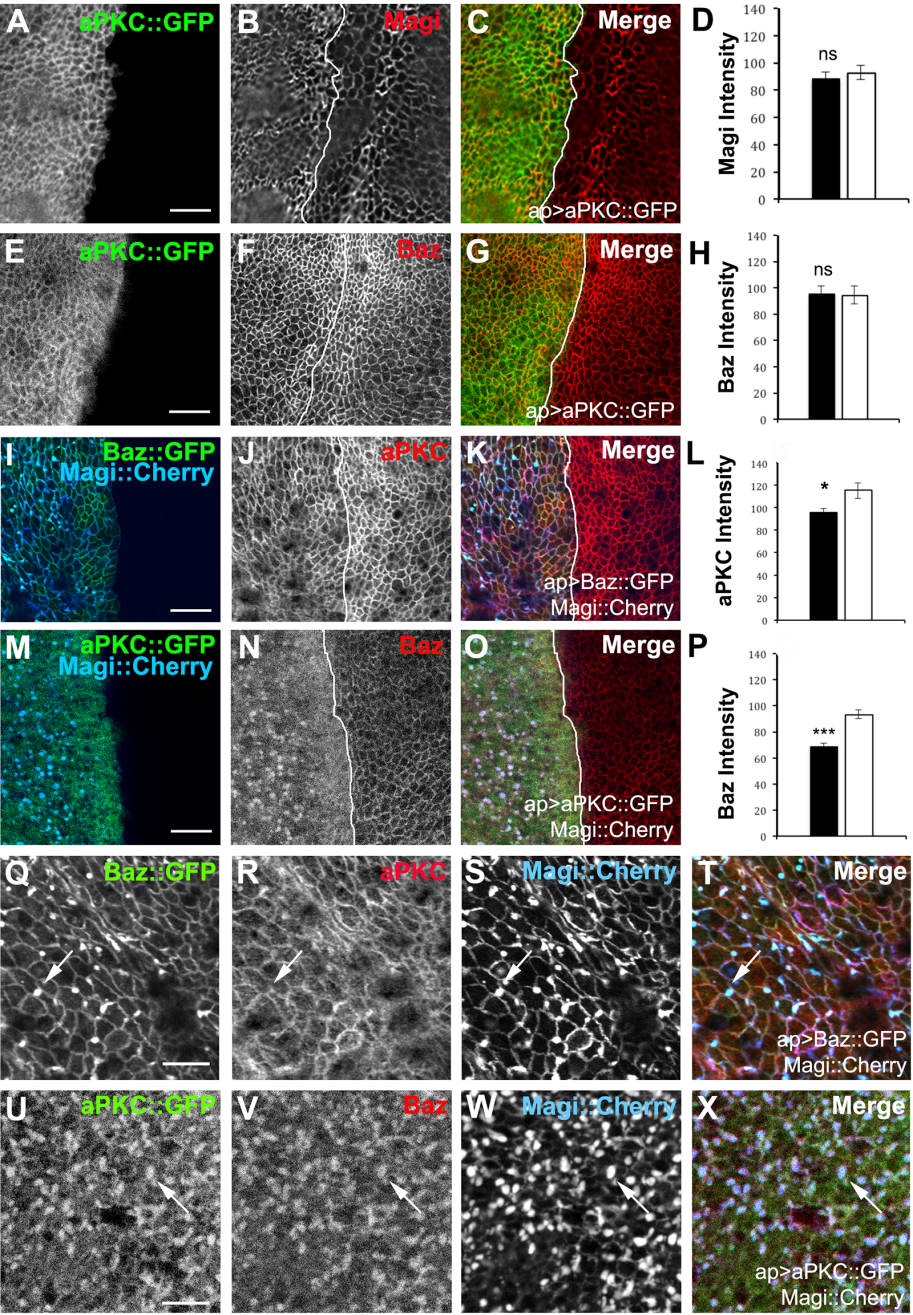
A balance between the levels of Magi and Baz regulates aPKC membrane levels. apterous-GAL4 was used to overexpress different combinations of tagged proteins in the wing imaginal disc. The average membrane intensity of each protein was measured and compared between the apterous (black bars) and non-apterous (white bars) side of the wing imaginal disc. The white lines mark the apterous dorsal/ventral boundary. (A-H) Overexpression of aPKC::GFP (green) had no effect on the membrane localization or levels of Magi (red) (A-D) or Baz (red) (E-H). (I-L) Co-expression of Magi::Cherry (blue) and Baz::GFP (green) attenuated the changes in aPKC (red) caused by high levels of Baz or Magi alone. aPKC levels were not increased by overexpression of Baz::GFP when Magi::Cherry was co-expressed. (M-P) Coexpression of Magi::Cherry (blue) and aPKC::GFP (green) lead to a reduction in the Baz membrane levels (red) and increased accumulation in the Magi vesicles. Overexpression of wildtype aPKC did not block the Magi induced reduction in Baz. (Q-X) Higher resolution image of the large accumulations seen with Magi::Cherry expression at the basolateral region of the epithelial cells. Each panel was digitally magnified 200%. (Q-T) Co-expression of Magi::Cherry (blue) and Baz::GFP (green) lead to the accumulation of Baz and Magi within the large internal accumulations. aPKC (red) was only weakly recruited (arrows). (U-X) Co-expression of Magi::Cherry (blue) and aPKC::GFP (green) lead to an increased accumulation of Magi vesicles that were also positive for Baz (red) (arrows). *** p<0.001; * p<0.05; ns not significant. Error bars indicate SEM. n = 5 discs for each experiment. Scale bars indicate 5μm for A-O and 2μm for Q-X.

Our results suggest a model in which Baz and Magi compete with each other or negatively regulate each other when expression levels are high. If this model was correct, we predicted that simultaneous overexpression of Magi with Baz should prevent excess Baz from interacting with apical polarity proteins and the subsequent recruitment of the apical polarity complex. We focused on the levels of aPKC to test this idea. Neither Magi nor Baz levels were affected when wildtype aPKC was overexpressed within the columnar epithelia (Fig 8A–8H) suggesting that increased aPKC levels per se are not affecting the recruitment of Magi. However, when both Baz and Magi were co-expressed, we found the increased levels of Magi in cells overexpressing Baz were able to block the increase of aPKC seen with overexpression of Baz alone (Fig 8I–8L). This result can also be viewed as Baz blocking the reduction of aPKC in Magi-overexpressing cells (compare with Fig 5I–5K), consistent with a Magi-Baz competition model. Simultaneous expression of aPKC and Magi resulted in a reduction of Baz at the membrane to a degree similar to Magi overexpression alone (Fig 8M–8P; compare with Fig 5A–5C).

Baz and Magi co-expression also resulted in their colocalization to the large internal puncta, while aPKC was excluded (Fig 8Q–8T). When both Magi and wildtype aPKC were overexpressed the number of large puncta greatly increased with spread into the apical domain and all three proteins, Magi, Baz and aPKC were found in the accumulations (Fig 8U–8X). These observations suggest that the loss of Baz or aPKC in the presence of excess Magi maybe due to the displacement of the polarity proteins to an intracellular domain. These results suggest that Magi and Baz have an antagonistic relationship and are perhaps in competition for a common binding partner or protein complex.

## Discussion

PDZ domain-containing proteins form scaffolding protein complexes with a wide range of roles including cell polarity and signaling. As a MAGUK protein, Magi is part of a scaffold that interacts with members of the polarity complex at the adherens junctions in the epithelia of the imaginal disc. The scaffolding function of Magi has been well established in other systems. In vertebrates epithelial cells MAGI-1 has been shown to act as structural scaffold at tight junctions [8,9] and adherens junctions [12,14]. In *C*. *elegans*, Magi-1 localizes apical to adherens junction and functions as an organizer to ensure that different cell adhesion complexes are segregated into distinct membrane domains along the lateral plasma membrane [21,22]. In neuronal cells MAGI-2/S-SCAM was also shown to cluster the cell adhesion molecule Sidekick, and the AMPA and NMDA glutamate receptors at the synapse [63–66].

Given the strong conservation of the Magi protein it is surprising that null mutants of *Drosophila Magi* exhibit no lasting cellular defects (other than transient defects in the interommatidial cells of the pupal eye [23]) and null animals are fully viable. Similarly in *C*.*elegans*, *magi-1* null worms are healthy with only a few embryos (1.3%) with defects during the ventral enclosure stage [21]. As Magi is highly conserved, it is plausible that Magi may only act in response to cell stress, DNA damage or some other trigger. For example, loss of p53 does not disrupt cellular function under normal conditions and p53 null flies or mice are viable with no cellular defects [67–69]. However, the role of p53 in response to DNA damage is well established and when these animals are exposed to irradiation apoptosis is not induced [67,70,71]. Alternatively, Magi function might be redundant with other components of the apical polarity complex or another protein and that loss of both is necessary for the disruption of cellular function. Core scaffolding components of the apicobasal polarity complex are dispensable for maintaining polarity in the wing imaginal disc epithelia supporting the idea of redundancy in this system. For instance, somatic clones of loss of function mutations in *crb*, *sdt* and *baz* have no effect on the polarity in the wing disc epithelia of the 3rd instar larvae [72,73]. Baz is a strong candidate for redundancy with Magi given the localization to the adherens junction and function as a PDZ scaffolding protein. As loss of *baz* in the wing imaginal disc does not disrupt the polarity of wing disc epithelia this leads to the hypothesis that Baz and Magi are redundant. However, somatic clones of a *baz* null mutant in a *Magi* mutant background did not lead to a loss of cell polarity or apoptosis (S2 Fig). While the two scaffolding proteins do not appear to functionally interact, we observed that Magi and Baz are in a protein complex and their close proximity within the wing columnar epithelia also suggests a common complex. Overexpression of Magi displaces Baz and aPKC from the apical membrane and, likewise overexpression of Baz displaces Magi from the membrane. The simultaneous over-expression of Magi and Baz suppresses the changes caused by their individual expression, suggesting a balance or competition between the two proteins. The maintenance of a balance between Magi and Baz might be due to a direct physical competition between these two proteins or opposite effects on a common mediator or interactor.

Baz and vertebrate MAGI proteins bind the lipid phosphatase PTEN and thus the Magi-Baz interaction and balance could be influenced by changes in the level of phosphoinositides such as PtdIns(4,5)P2 (PIP2) or PtdIns(3,4,5)P3 (PIP3). In polarized epithelia, PIP2 is found within the apical domain and PIP3 restricted to the basal-lateral domain [74]. Baz localization in polarized epithelia depends on PIP2 and on the PI4P5 kinase Skittles [59–61]. Baz in turn can be a positive regulator of PIP2 levels at the plasma membrane by local recruitment of the lipid phosphatase PTEN [62,75]. We observed an increase in PIP3 levels with increased expression of Magi, which may reflect the loss of Baz and a loss of PTEN recruitment to the membrane. We were not able to assess changes in PTEN levels at the membrane with available antibodies. However we did observe that the recruitment of Magi or Baz was not affected in *Pten* mutant cells. Similarly the changes in PIP3 levels are unlikely to be the cause of Baz loss in the presence of increased Magi as co-expression of PTEN and Magi still resulted in the loss of Baz from the membrane. Prior studies on Magi in *Drosophila* in the pupal eye did not detect any physical interaction between *Drosophila* Magi and Pten, and the phenotypes generated by overexpression of Magi in the *Drosophila* eye were not affected by *Pten* mutants [23]. Therefore it is likely that loss of Baz in the presence of increased Magi in the wing imaginal disc and vice versa is through competition for a protein component.

In the developing eye Magi forms a protein complex with RASSF8 (the N-terminal Ras association domain-containing protein) and ASPP (Ankyrin-repeat, SH3-domain, and proline-rich-region containing protein), and this complex plays a role during remodeling of the adherens junctions in the interommatidial cells (IOCs) [23]. When IOCs rearrange to create the pupal lattice, this process requires regulation of the E-Cadherin complex where RASSF8 and ASPP regulate adherens junction remodeling and integrity through regulation of Src kinase activity [76,77]. Magi recruits the RASSF8-ASPP complex in the process of adherens junction remodeling and there are defects in IOC rearrangement in *Magi* mutants where AJs are frequently interrupted [23]. In the eye the Magi-RASSF8-ASPP complex is necessary for the cortical recruitment of Baz and of the adherens junction proteins α- and β-catenin. A model has been proposed where Magi-RASSF8-ASPP complex functions to localize Baz to remodeling junctions to promote the recruitment or stabilization of E-Cad complexes [23]. However, we do not think that the RASSF8-ASPP complex is the point of competition between Magi and Baz within the wing imaginal disc. In the wing imaginal disc Magi and the RASSF8-ASPP complex are localized to the adherens junction domain independently [23] and while *RASSF8* mutants have a wing rounding phenotype [78], *Magi* mutants do not (this work and [23]). Furthermore we observed no differences in Baz, Ecad or Arm distribution in *Magi* somatic loss of function clones in the wing imaginal disc. Finally the Magi WW domains are required for the interaction with RASSF8 [23], while the overexpression of the Magi transgene that contains the PDZ domains led to a reduction in Baz suggesting that second half of the Magi protein containing the PDZ domains contains the important sites for this competition.

Therefore, a strong possibility to explain the reciprocal effects of overexpression is that Baz and Magi compete for a common binding site. We found that Magi interacted with both Baz and aPKC; the latter two are known to interact directly [79,80]. However, it is unlikely that the shared site is through physical scaffolding of aPKC, as high levels of wild type aPKC had no effect on either Magi or Baz and was not able rescue the changes in Baz levels and localization caused by Magi overexpression. In addition the overexpression of Magi also led to a reduction in aPKC. It is unlikely that the loss of Baz is responsible for this displacement as aPKC is not mislocalized in Baz clones and Baz is not mislocalized in *Par-6*, *aPKC* or *Cdc42* null clones [81,82]. Further investigation is required to explore the mechanisms that underlie Magi interactions with components of the apical polarity complex and the adherens junction complex.

## Supporting Information

S1 Fig Magi protein, gene and *Magi*^*bst*^ structure. (A) Diagram of the Magi proteins from *Caenorhabditis elegans*, *Drosophila melanogaster* and vertebrates (human). All homologues contain multiple PDZ domains (blue) and two WW domains (orange) while the GUK domain (green) and the 5’ most PDZ domain is missing from the invertebrate genes. (B) Western blot showing the specificity of Magi antibody and that the *Magi*^*bst*^ allele is a null allele. Western blots of embryonic and third instar wing imaginal discs extracts with the Magi antibody detected a band of 130 KDa in WT extracts, which was absent in *Magi*^*bst*^ extracts. A nonspecific band of lower molecular weight was present in all extracts and likely corresponds to the non-specific nuclear epitope that is still observed in *Magi* mutant cells. Anti-Tubulin was used as loading control. (C) Diagrams showing Magi gene structure and its three different isoforms as well as the *Magi*^*bst*^ allele. Gene orientation is 3’ to 5’ to match the orientation in FlyBase. *Magi*^*bst*^ is a 8.5 kb deletion between the first and last exons of the *Magi* gene and removes the two WW domains (green boxes) and the first three PDZ domain (blue boxes). The antibody to Magi is to the C-terminal end (arrow).
(TIF)

S2 Fig Cells mutant for both *baz* and *Magi* are viable with no loss of polarity. (A-D) *baz*^*4*^ mutant clone in a homozygous *Magi*^*bst*^ mutant wing disc. Magi (blue), Baz (red) and GFP (green). Cells in the somatic FRT mediated clone (black area) are double mutants for *baz* and *Magi* and are viable. Note the nuclear labeling with the Magi antibody represents an unrelated epitope.
(TIF)

S3 Fig The effect of overexpressed Magi is specific to the apical polarity complex. Wing imaginal discs overexpressing Magi::Cherry (red) using apterous-Gal4. For each protein the average intensity of immunolabeling was measured on the apterous (black bars) versus non-apterous (white bars) side of the wing imaginal disc and plotted. The white lines mark the apterous dorsal/ventral boundary. (A-D) Overexpression of Magi resulted in reduction in the membrane level of Par-6 (green). Par-6 did not co-localize to the large Magi accumulations (arrow). (E-H). High levels of Magi reduced the level of Crb (green) at the plasma membrane. (I-L). Overexpression of Magi had no effect on the adherens junction protein Ecad (green). (M-P). Overexpression of Magi had no effect on the basolateral polarity protein Dlg (green). Statistical significance on the plots is indicated with asterisks. *** p<0.001; ns p>0.05. Error bars indicate SEM. Scale bars indicate 5μm.
(TIF)

S4 Fig Changes in Dlg and PTEN do not alter Baz or Magi levels or localization. (A-H) Wing imaginal discs overexpressing different tagged proteins using apterous-Gal4. For each protein assayed the average intensity of immunolabeling was measured on the apterous (black bars) versus non-apterous (white bars) side of the wing imaginal disc and plotted. White lines indicate the apterous boundary. (A-D) Overexpression of Baz::GFP (green) had no effect on the basolateral polarity protein Dlg (red). (E-H) Overexpression of Dlg::GFP (green) did not alter the membrane localization and levels of Magi (red). Statistical significance on the plots is indicated with asterisks. ns p>0.05. Error bars indicate SEM. n = 5 discs for each experiment. Scale bars indicate 5μm. (I-N) Somatic clones of a *Pten* mutant had no effect on Magi or Baz. FRT mediated somatic clones of *Pten*^*dj189*^ (I, L, non-GFP) were immunolabeled for Magi (J, red) or Baz (M, red). Localization and levels of Magi and Baz were not altered.
(TIF)
