# Supplementary figures and images for: Magi Is Associated with the Par Complex and Functions Antagonistically with Bazooka to Regulate the Apical Polarity Complex
Source: PLoS One. 2016 Apr 13;11(4):e0153259. doi: 10.1371/journal.pone.0153259 (PMC4830575; doi:10.1371/journal.pone.0153259)

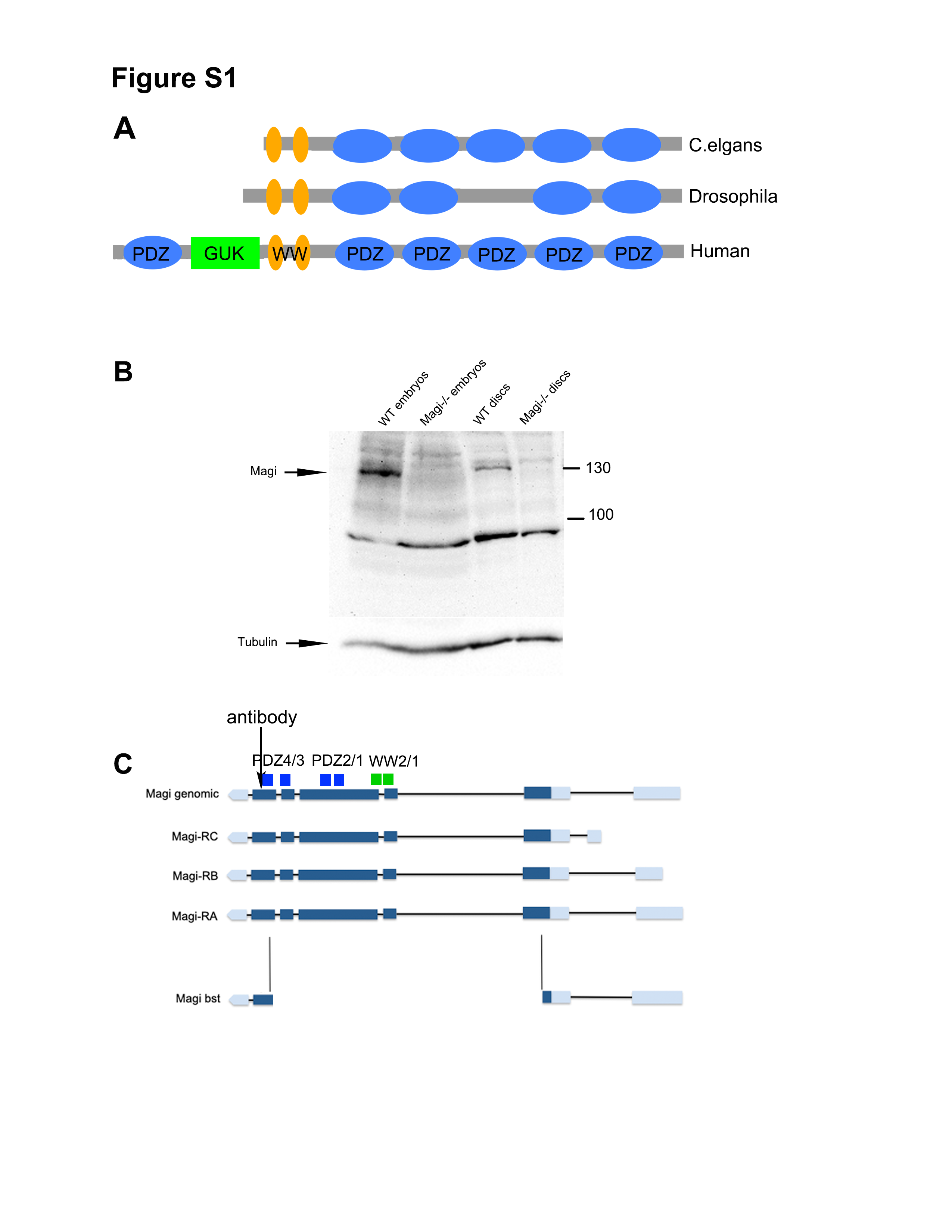

Supplement: S1 Fig — (A) Diagram of the Magi proteins from Caenorhabditis elegans, Drosophila melanogaster and vertebrates (human). All homologues contain multiple PDZ domains (blue) and two WW domains (orange) while the GUK domain (green) and the 5’ most PDZ domain is missing from the invertebrate genes. (B) Western blot showing the specificity of Magi antibody and that the Magibst allele is a null allele. Western blots of embryonic and third instar wing imaginal discs extracts with the Magi antibody detected a band of 130 KDa in WT extracts, which was absent in Magibst extracts. A nonspecific band of lower molecular weight was present in all extracts and likely corresponds to the non-specific nuclear epitope that is still observed in Magi mutant cells. Anti-Tubulin was used as loading control. (C) Diagrams showing Magi gene structure and its three different isoforms as well as the Magibst allele. Gene orientation is 3’ to 5’ to match the orientation in FlyBase. Magibst is a 8.5 kb deletion between the first and last exons of the Magi gene and removes the two WW domains (green boxes) and the first three PDZ domain (blue boxes). The antibody to Magi is to the C-terminal end (arrow). (TIF) [file pone.0153259.s001.tif]

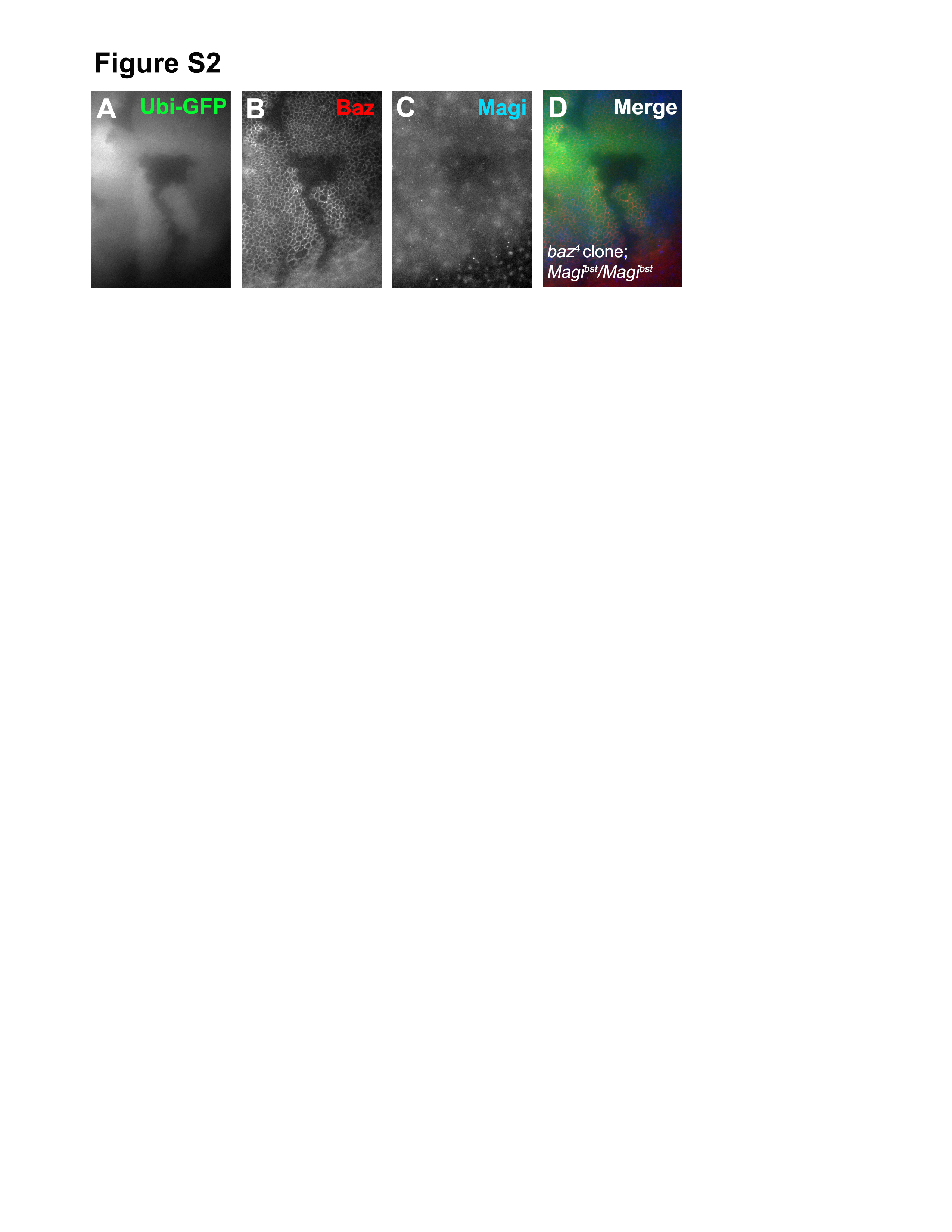

Supplement: S2 Fig — (A-D) baz4 mutant clone in a homozygous Magibst mutant wing disc. Magi (blue), Baz (red) and GFP (green). Cells in the somatic FRT mediated clone (black area) are double mutants for baz and Magi and are viable. Note the nuclear labeling with the Magi antibody represents an unrelated epitope. (TIF) [file pone.0153259.s002.tif]

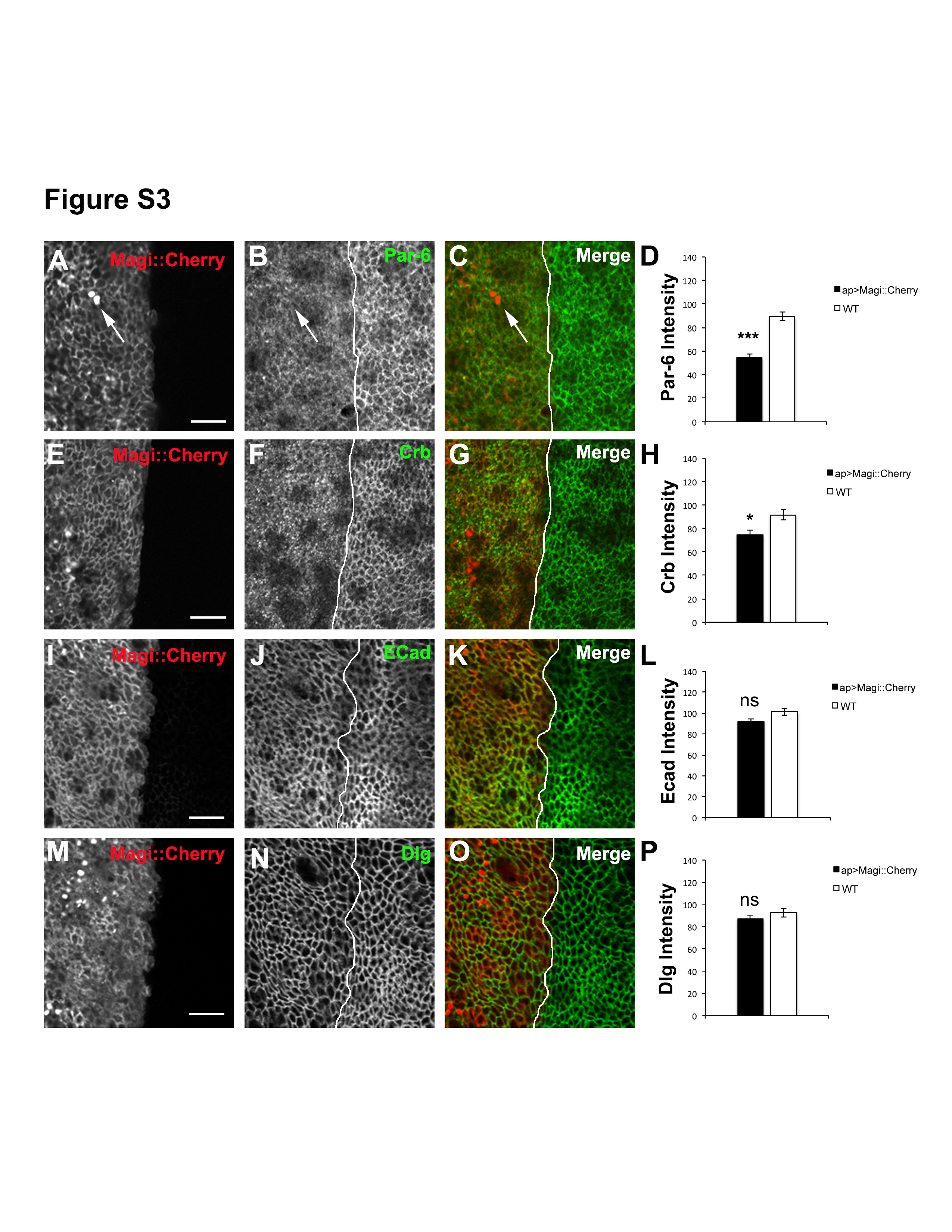

Supplement: S3 Fig — Wing imaginal discs overexpressing Magi::Cherry (red) using apterous-Gal4. For each protein the average intensity of immunolabeling was measured on the apterous (black bars) versus non-apterous (white bars) side of the wing imaginal disc and plotted. The white lines mark the apterous dorsal/ventral boundary. (A-D) Overexpression of Magi resulted in reduction in the membrane level of Par-6 (green). Par-6 did not co-localize to the large Magi accumulations (arrow). (E-H). High levels of Magi reduced the level of Crb (green) at the plasma membrane. (I-L). Overexpression of Magi had no effect on the adherens junction protein Ecad (green). (M-P). Overexpression of Magi had no effect on the basolateral polarity protein Dlg (green). Statistical significance on the plots is indicated with asterisks. *** p<0.001; ns p>0.05. Error bars indicate SEM. Scale bars indicate 5μm. (TIF) [file pone.0153259.s003.tif]

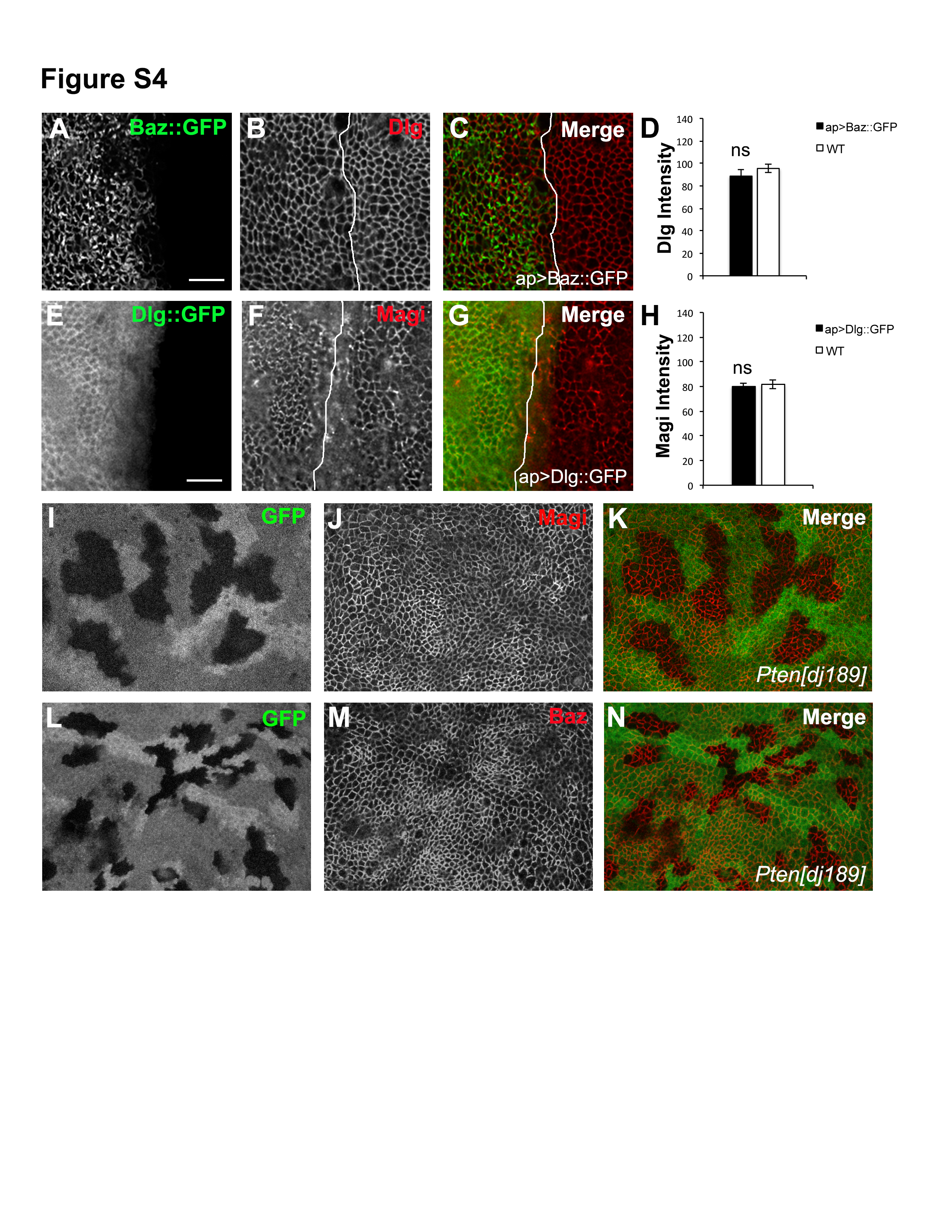

Supplement: S4 Fig — (A-H) Wing imaginal discs overexpressing different tagged proteins using apterous-Gal4. For each protein assayed the average intensity of immunolabeling was measured on the apterous (black bars) versus non-apterous (white bars) side of the wing imaginal disc and plotted. White lines indicate the apterous boundary. (A-D) Overexpression of Baz::GFP (green) had no effect on the basolateral polarity protein Dlg (red). (E-H) Overexpression of Dlg::GFP (green) did not alter the membrane localization and levels of Magi (red). Statistical significance on the plots is indicated with asterisks. ns p>0.05. Error bars indicate SEM. n = 5 discs for each experiment. Scale bars indicate 5μm. (I-N) Somatic clones of a Pten mutant had no effect on Magi or Baz. FRT mediated somatic clones of Ptendj189 (I, L, non-GFP) were immunolabeled for Magi (J, red) or Baz (M, red). Localization and levels of Magi and Baz were not altered. (TIF) [file pone.0153259.s004.tif]
